# Towards Generative Design of Computationally Efficient Mathematical Models with Evolutionary Learning

**DOI:** 10.3390/e23010028

**Published:** 2020-12-27

**Authors:** Anna V. Kalyuzhnaya, Nikolay O. Nikitin, Alexander Hvatov, Mikhail Maslyaev, Mikhail Yachmenkov, Alexander Boukhanovsky

**Affiliations:** Nature Systems Simulation Lab, National Center for Cognitive Research, ITMO University, 49 Kronverksky Pr., 197101 St. Petersburg, Russia; nnikitin@itmo.ru (N.O.N.); alex_hvatov@itmo.ru (A.H.); mikemaslyaev@itmo.ru (M.M.); mmiachmenkov@itmo.ru (M.Y.); boukhanovsky@mail.ifmo.ru (A.B.)

**Keywords:** generative design, automated learning, evolutionary learning, co-design, genetic programming

## Abstract

In this paper, we describe the concept of generative design approach applied to the automated evolutionary learning of mathematical models in a computationally efficient way. To formalize the problems of models’ design and co-design, the generalized formulation of the modeling workflow is proposed. A parallelized evolutionary learning approach for the identification of model structure is described for the equation-based model and composite machine learning models. Moreover, the involvement of the performance models in the design process is analyzed. A set of experiments with various models and computational resources is conducted to verify different aspects of the proposed approach.

## 1. Introduction

Nowadays, data-driven modeling is a very popular concept, first of all because of many examples of the successful application for a wide range of tasks where we have data samples which are sufficient for model training. However, originally the term “modeling” assumes a wider meaning than just identifying numerical coefficients in equations. One may say that modeling is an art of creation of mathematical (in the context) models that describe processes, events, and systems with mathematical notation. And current successes of artificial intelligence (AI) give the opportunity to come closer to the solution of the task of mathematical modeling in this original formulation.

For this purpose we may use an approach of generative design that assumes open-ended automatic synthesis of new digital objects or digital reflections of material objects which have desired properties and are aligned with possible restrictions. Open-ended evolution is a term that assumes ongoing generation of novelty as new adaptations of specimens, new entities and evolution of the evolvability itself [1]. We assume that new objects are objects with essentially new features that appeared during the adaptation process and that can’t be obtained with simple tuning or recombination of initially known parameters. Other words, it is an approach that aims of algorithmic “growing” of a population of new objects when each of them is aligned with restrictions and have desired properties, to some extent. However, only the objects which could maximize the measure of fitness will be used for their intended purpose. The generative design is a well-known concept for creation of digital twins of material objects [2]. The same idea can be applied to mathematical models [3]. Indeed, it is known that we may grow mathematical expressions that approximate some initial data with a symbolic (usually polynomial) regression approach. However, if we look at mathematical expressions in a wider perspective we may admit that expressions could be different even much more complicated. For example, we may try to apply this approach to the problem of searching for an equation of mathematical physics that is able to describe observed phenomena. Or, we may want to create in an automated way a complicated data-driven model that consists of many single models and feature processing stages. Tasks in both examples can be formalized as the generative design of computer models.

Both of cases (model as mathematical equation and complicated data-driven models) have their own spheres of application, but they also can be joined as composite models. In machine learning the composite model case often is described in terms of the multi-model data-driven pipelines. If a single data-driven model cannot provide appropriate results, various ensembling techniques like stacking or blending are applied [4]. To achieve better quality, complex modeling pipelines can be used, that include different pre-processing stages and can contain several types of models. A generalization of ensembling approaches is the composite model concept [5]. A composite model has a heterogeneous structure, so it can include models of different nature: machine learning (ML), equation-based, etc. [6].

A design of a composite model can be represented from an automated ML (AutoML) perspective that may use a genetic algorithm for learning the structure. The evolutionary learning approach seems to be a natural and justified choice because of several reasons. First of all, the idea of generative design refers to the possibility of controlled open-ended evolution under a set of restrictions. After that, genetic algorithms give flexible opportunities for treating mixed problems with combinatorial and real parts of a chromosome.

**Figure 1:**
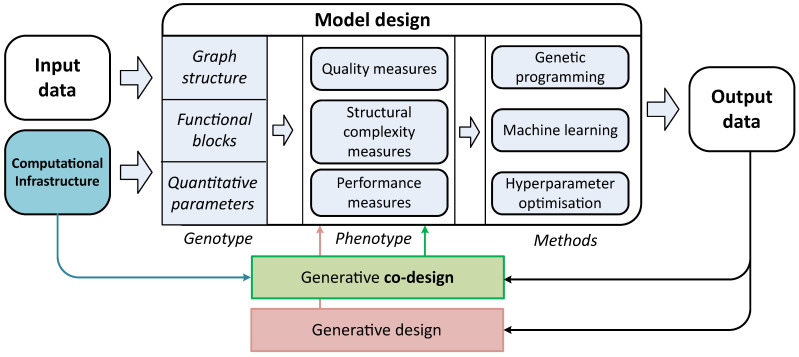
The description of the generative co-design concept: the different aspects of the model design (genotype, phenotype, and the identification methods); the pipeline of the data-driven modeling; the difference between classical design approach and co-design approach.

However, the design of the composite model may depend on different factors: the desired modeling quality, computational constraints, time limits, interpreting ability requirements, etc. It raises the problem of co-design [7] of the automatically generated composite models with the specific environment. Generative co-design is an approach which allows to synthesize jointly a set (mostly a pair) of objects that will be compatible with each other. In context of this article these are mathematical models and computational infrastructure. The conceptual difference between the generative design (that builds the model on a basis of dataset only) and the generative co-design (that takes into account both data and infrastructure) is illustrated in Figure 1. The structure of composite models can be very complex, so it is complicated to construct the models in an expert way. For this reason, different optimization techniques are used for the structural learning of the model. Usually, the objective function for optimization is aimed to minimize the error of the predictions obtained from the candidate model [8].

The paper is organized as follows. Section 2 describes the existing approaches to the design of models. Section 3 provides the mathematical formulation for the model’s design and co-design tasks and associated optimization problems. Section 4 described the actual issues of generative co-design for the mathematical models. Section 5 provides the results of experimental studies for different applications of generative design (composite models, equation-based models, etc). The unsolved problems of co-design and potential future works are discussed in Section 6. Section 7 provides the main conclusions.

## 2. Related Work

An extensive literature review shows many attempts for mathematical models design in the different fields [9,10]. In particular, the methods of the automated model design is highly valuable part of the various researches [11]. As an example, the equation-free methods allow building the models that represent the multi-scale processes [12]. Another example is building of the physical laws from data in form of function [13], ordinary differential equations system [14], partial differential equations (PDE) [15]. The application of the automated design of ML models or pipelines (which are algorithmicaly close notions) are commonly named AutoML [8] although most of them work with models of fixed structure, some give opportunity to automatically construct relatively simple the ML structures. Convenient notation for such purpose is representation of a model as a directed acyclic graph (DAG) [16]. Another example of popular AutoML tool for pipelines structure optimization is TPOT [17].

To build the ML model effectively in the complicated high-performance environment [18], the properties of both algorithms and infrastructure should be taken into account. It especially important for the non-standard infrastructural setups: embedded [19], distributed [20], heterogeneous [21] systems. Moreover, the adaptation of the model design to the specific hardware is an actual problem for the deep learning models [22,23].

However, the application of co-design approaches [24] for the generative model identification in the distributed or supercomputer environment [25,26] is still facing a lot of issues. For example, the temporal characteristics of the designed models should be known. The estimations of fitting and simulation time of the data-driven models can be obtained in several ways. The first is the application of the analytical performance models of the algorithm [27]. The identification of the analytical performance models can be achieved using domain knowledge [28]. However, it can be impossible to build this kind of model for the non-static heterogeneous environment. For this reason, the empirical performance models (EPMs) are highly applicable to the different aspects of the generative model design [29]. Moreover, the effective estimation of execution time is an important problem for the generation of optimal computational schedule [30] or the mapping of applications to the specific resources [31].

The execution of the complex resource-consuming algorithms in the specific infrastructure with limited resources raises the workflow scheduling problem [32]. It can be solved using an evolutionary algorithm [33] or neural approaches [34].

It can be noted that the existing design and co-design approaches are mostly focused on the specific application and do not consider the design for the different types of mathematical models. In the paper, we propose the modified formulation of this problem that allows applying the generative design and co-design approaches to the different tasks and models.

## 3. Problem Statement

A problem of the generative design of mathematical models requires a model representation as a flexible structure and appropriate optimization methods for maximizing a measure of the quality of the designed model. To solve this optimization problem, different approaches can be applied. The widely used approach is based on evolutionary algorithms (e.g., genetic optimization implemented in TPOT [35] and DarwinML [16] frameworks) because it allows solving both exploration and exploitation tasks in a space of model structure variants. The other optimization approaches like the random search of Bayesian optimization also can be used, but the populational character of evolutionary methods makes it possible to solve the generative problems in a multiobjective way and produce several candidates model. Such formulation also can be successfully treated with the evolutionary algorithms or hybrid ones that combine the use of evolutionary operators with additional optimization procedures for increasing of robustness and acceleration of convergence. In this section, we describe the problem of generative co-design of mathematical models and computational resources in terms of the genetic programming approach.

A general statement for numerical simulation problem can be formulated as follows:

(1) Y=H(M|Z),

where H is an operator of simulation with model *M* on data *Z*.

In the context of problem of computer model generative design, the model *M* should have flexible structure that can evolve by changing (or adding/eliminating) the properties of a set of atomic parts (“building blocks”). For such task, the model *M* can be described as a graph (or more precisely as a DAG):

(2) $$M=\langle S,E,\left\{a_{1:|A|}\right\}\rangle ,$$

with edges *E* that denoted relations between nodes $\langle S,\left\{a_{1:|A|}\right\}\rangle$ that characterize functional properties *S* of atomic blocks and set of their parameters $\left\{a_{1:|A|}\right\}$.

In terms of evolutionary algorithms each specimen $d_{p}$ in population *D* of computer model can be represented as a tuple that consists of phenotype *Y*, genotype *M* and fitness function $\varphi \left(M\right)$:

(3) $$d_{p}=\langle Y_{p},M_{p},\varphi \left(M_{p}\right)\rangle ,\;D=\;\left(d_{p},\;p\in \left[1:\left|D\right|\right]\right).$$

Genotype *M* should be mapped on plain vector as a multi-chromosome that consists of three logical parts: functional properties, sets of their parameters, relations between blocks:

(4) $$M_{p}=\langle S_{p},E_{p},{\left\{A_{k}\right\}}_{p}\rangle =\langle {\left\{s_{1:\left|S_{p}\right|}\right\}}_{p},{\left\{e_{1:\left|E_{p}\right|}\right\}}_{p},{\left\{a_{1:\left|S_{p}\right|\left|A_{k}\right|}\right\}}_{p}\rangle ,A_{k}={\left\{a_{1:\left|A_{k}\right|}\right\}}_{k},\;k\in \left[1:\left|S_{p}\right|\right].$$

**Figure 2:**
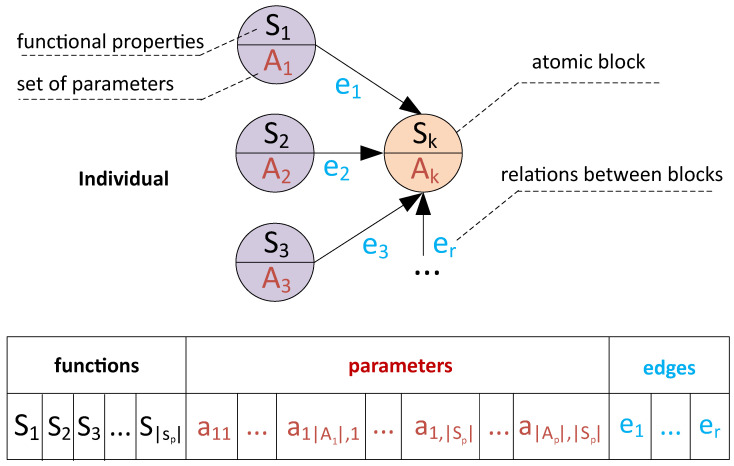
The structure of the genotype during evolutionary optimization: functional properties, set of parameters and relations between atomic blocks.

The genotype is also illustrated in Figure 2.

An important property is that $\left|S_{p}\right|,\left|A_{p}\right|,\left|E_{p}\right|\neq const$, what means varying overall size of chromosome (and its structure). Such property makes this approach is really open-ended and consistent with idea of model evolution because it give an opportunity to synthesize the models with truly new features instead of simple recombination and optimization of existed ones. Technically open-endedness here refers to the ability of generative design algorithms to expand or narrow a combinatorial search space in the process of optimization with evolutionary operators. This leads to need of special realizations for crossover and mutation operators. As the chromosome $M_{p}$ is a ordered set with the structure fixed in a tuple $\langle S,\left\{a_{1:|A|}\right\},E\rangle$ it is necessary to preserve this structure after crossover and mutation. That’s why these operators are written relative to the graph structure and influence on the parts of chromosome that describe the node or a set of nodes with associated edges (sub-graphs). We may say that each single node can be described as some function with parameters $y_{k}=f_{k}\left(x,{\left\{a_{1:|A|}\right\}}_{k}\right)$. And mutation of function $f_{k}$ performs symbolic changes in the mathematical expression that results in extension of range of limits of initial genes.

So, the task of mathematical model generative design can be formulated as optimization task:

(5) $${p_{Q}}^{max}\left(M^{*}\right)=\max_{M}\;f_{Q}\left(M|I^{+},T_{gen}\leq \tau_{g}\right),\;\;\;M=\;\left\{M_{p}\right\},$$

where $f_{Q}$ is a fitness function that characterizes quality generated mathematical and ${p_{Q}}^{max}$ is a maximal value of fitness function, model *M* is a space of possible model structures, $I^{+}$ - actual computational resources, $T_{gen}$ is time for model structure optimization critical threshold $\tau_{g}$. In such formulation we try to design the model with the highest quality, but we need to rely optimization to single configuration of computational resources. This factor is a strong limitation for the idea of generative design because this idea assumes flexibility of searched solution including the possibility to find the most appropriate for applied task combination of model structure and computational resources. The concept was illustrated on Figure 1.

Model and computational infrastructure co-design may be formulated as follows:

(6) $$p^{max}\left(M^{*},I^{*}\right)=\max_{M,I}\;F\left(M,I|T_{gen}\leq \tau_{g}\right),\;\;I=\;\left\{I_{q}\right\},\;\;M=\;\left\{M_{p}\right\},$$

where *I* is a set of infrastructure features, *F* is a vector fitness function that characterize a trade off between a goodness of fit and computational intensity of model structure. Vector function *F* consists of quality function $f_{Q}$ and time function $f_{T}$ that is negative for correct maximization:

(7) $$F\left(M,I\right)\;=\;\left(f_{Q}\left(M,I\right),-f_{T}\left(M,I\right)\right).$$

**Figure 3:**
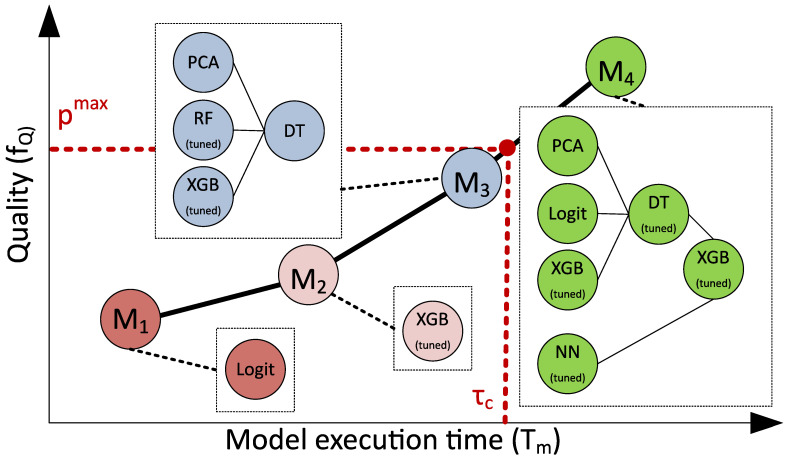
Pareto frontier obtained after the evolutionary learning of the composite model in the “quality-execution time” subspace. The points referred as $M_{1}$–$M_{4}$ represent the different solutions obtained during optimization. $p^{max}$ and $\tau_{c}$ represent quality and time constraints.

The time function $f_{T}$ is a function that shows expected execution time of the model that is being synthesized with generative design approach. As the model *M* is still in the process of creation at the moment we want to estimate *F*, the values of $f_{T}$ may be defined by performance models (e.g., Equation (9)). The example of the model selection from the Pareto frontier on a basis of $p^{max}$ and $\tau_{c}$ constraints is presented is Figure 3. It can be seen that model $M_{4}$ has the better quality but it does not satisfy the execution time constraint $\tau_{c}$.

However, in most of cases correct reflection of infrastructure properties to model performance is quite complicated task. In described case when we need, first, to generate the model with appropriate quality and vital limitations for computation time, we have several issues: (1) we may be not able to estimate model performance with respect to certain infrastructure in straight forward way and as a consequence we need performance models; (2) estimation of the dependency between model structure and computational resources reflects only mean tendency due to number of simplifications in performance models and search for minima on such uncertain estimations lead to unstable convergence to local minima. Due to these issues the formulation of optimization for co-design on stage of model building may be simplified to single criteria problem $F\left(M,I|T_{gen}\leq \tau_{g}\right)\;\approx \;\;F^{\prime }\left(M|\;\;T_{M}\leq \tau_{},\;T_{gen}\leq \tau_{g}\right)$ with change of direct usage of infrastructure features to estimated time of model execution via performance models $\;\;T_{M}\;\;\approx T=\;f_{T}\left(M,I\right)$:

(8) $${\hat{p}}^{max}\left(M^{*}\right)\;=\max_{M}\;{\hat{f}}_{Q}\left(M|\;\;T_{M}\leq \tau_{c},\;T_{gen}\leq \tau_{g}\right),$$

where $f_{Q}$ is single criteria fitness function that characterize goodness of fit of model with additional limitations for expected model execution time $\;T_{M}$ and estimated time for structural optimization $T_{gen}$.

In the context of automated models building and their co-design with computational resources, performance models (PM) should be formulated as a prediction of expected execution time with the explicit approximation of a number of operations as a function of computer model properties $S,\left\{a_{1:\left|S\right|}\right\}$ and infrastructure *I* parameters. However, for different computer models classes, there are different properties of performance models. In the frame of this paper, we address the following classes of models: ML models, numerical models (based on the numerical solution of symbolic equations), and composite models (that may include both ML and numerical models).

For ML models PM can be formulated as follows:

(9) $$T_{\mathrm{ML}}^{\mathrm{PM}}\left(Z,M\right)\;=max_{i}\left[\sum_{it}\;\frac{OML_{i,it}}{V_{i}\left(I\right)+Const_{i}\left(I\right)}\;\right]+O\left(I\right),$$

where $OML=OML\left(Z,M\right)$ is an approximate number of operations for data-driven model with data volume *Z* and parametric model *M*, it—iterator for learning epoch, $V_{i}\left(I\right)$ is for performance of $i^{\prime }th$ computational node in flops, $Const_{i}\left(I\right)$ is for constant overheads for $i^{\prime }th$ node in flops, $O\left(I\right)$ is for sequential part of model code.

According to structure $M=\langle S,E,\left\{a_{1:\left|S\right|}\right\}\rangle$ for data driven-model case, duple $\langle S,E\rangle$ characterize structural features of models (e.g., activation functions and layers in neural networks) and $\left\{a_{1:\left|S\right|}\right\}$ characterize hyper-parameters.

For numerical models PM can be formulated as follows:

(10) $$T_{Num}^{\mathrm{PM}}\left(R,M\right)=max_{i}\left[\frac{ON_{i}}{V_{i}\left(I\right)+Const_{i}\left(I\right)}\right]+O\left(I\right),$$

where $ON=ON\left(R,M\right)$ is an approximate number of operations for numerical model. In distinction with ML models they are not required for learning epochs and do not have strong dependency from volume of input data. Instead of this, there are internal features of model *M*, but it is worth separately denote computational grid parameters *R*. They include parameters of grid type, spatial and temporal resolution. Among the most influential model parameters *M* there are type and order of equations, features of numerical solution (e.g., numerical scheme, integration step, etc.).

For composite models PM total expected time is a sum of expected times for sequential parts of model chain:

(11) $$T_{Comp}^{\mathrm{PM}}\left(R,Z,M\right)=\;\sum_{j}\;max_{i}\left[\frac{OC_{i,j}}{V_{i}\left(I\right)+Const_{i}\left(I\right)}\right]+O\left(I\right),$$

where expected time prediction for each sequential part is based on properties of appropriate model class:

(12) $$OC=\;\left\{\begin{array}{c} OML,\;\;if\;model\;is\;ML\;\\ ON,\;\;if\;model\;is\;numerical\; \end{array}\right..$$

## 4. Important Obstacles on the Way of Generative Co-Design Implementation

It may seem that the problem statement described above gives us a clear vision of an evolutionary learning approach for generative design and co-design. However, several subtle points should be highlighted. This section is devoted to a discussion of the most interesting and challenging points (in the authors’ opinion) that affect the efficiency or even the possibility of implementation the generative design (and co-design) approach for growing new mathematical models.

*Issue 1. Numerical Methods for Computation of Designed Arbitrary Function*

Open-ended realization of automated symbolic model creation with a generative design approach leads to the possibility of getting an unknown function as a resulted model. On the one hand, it gives interesting perspectives to create the new approximations of unknown laws. However, on the other hand, this possibility leads to the first conceptual problem of the generative design of mathematical models and a serious stumbling block on the way to implementing this idea. This problem is the need to calculate an arbitrary function or get the numerical solution of an arbitrary equation.

The choice of the numerical method for a given problem (discovered algebraic, ordinary differential, partial differential equation equations) is the crucial point. In most cases, the numerical method is designed to solve only several types of equations. When the numerical method is applied to the problem type, where convergence theorem is not proved, the result may not be considered as the solution.

As an example, solution of the partial difference equations using the finite difference schemes. For brevity, we omit details and particular equations, the reader is referred to [36] for details. The classical one-dimensional diffusion equation has different schemes, in particular, explicit, implicit, Crank-Nicolson scheme. Every scheme has a different approximation order and may lead to different solutions depending on the time-spatial grid taken. If the Crank-Nicolson spatial derivative scheme is taken to solve another equation, for example, the one-dimensional string equation, then the solution will also depend on the time-spatial grid taken, however, in another manner. It leads to the general problem that the particular finite schemes cannot be used for the general equation solution.

The second approach is to approximate a solution with a neural network, which somewhat mimics the finite element method. The neural networks are known as universal approximators. However, their utility for differential equations solution is still arguable. The main problem is that the good approximation of the field is not necessary leads to the good derivative approximation [37]. There is a lot of workarounds to approximate derivatives together with the initial field, however, it is done with the loss of generality.

The possible promising solution is to combine optimization methods, local neural network approximation, and classical approach [38]. However, there is still a lot of the “white spots”, since the arbitrary equation means a strongly non-linear equation with arbitrary boundary conditions. Such a generality cannot be achieved at the current time and requires a significant differentiation, approximation, and numerical evaluation method development. The illustration examples of the inverse problem solution are shown in Section 5.1.

*Issue 2. Effective Parallelization of Evolutionary Learning Algorithm*

The procedure of generative design has high computation cost, thus effective algorithm realization is highly demanded. Efficiency can be achieved primarily by parallelizing the algorithm. As discussed generative algorithm is implemented on a base of the evolutionary approach, so the first way is a computation of each specimen $d_{p}$ in a population in a separate thread. Strictly speaking, it may be not only threads, but also separate computational nodes for clusters, but not to confuse computer nodes with nodes of a model graph $M_{p}$, here and further we will use the term “thread” in a wide sense. This way is the easiest for implementation but will be effective only in the case of cheap computations of objective function $\varphi \left(M\right)$.

The second way is acceleration of each model $M_{p}$ on the level of its nodes $\langle S,\left\{a_{1:\left|A\right|}\right\}\rangle$ with possibility of logical parallelized. However, this way seems to be the most effective if we have uniform (from the performance point of view) nodes of models $M_{p}$ and computational intensity appropriate for used infrastructure (in other words, each node should be computed in a separate thread in acceptable time). Often for cases of composite models and numerical models, this condition is becoming violated. Usually, the numerical model is consists of differential equations that should be solved on large computational grids. And composite models may include nodes that are significantly more computationally expensive than others. All these features lead us to take into account possibility of parallelization of generative algorithm on several levels: (1) population level, (2) model $M_{p}$ level, (3) each node $\langle S,\left\{a_{1:\left|A\right|}\right\}\rangle$ level; and make an adaptation of algorithm with respect to certain task.

**Figure 4:**
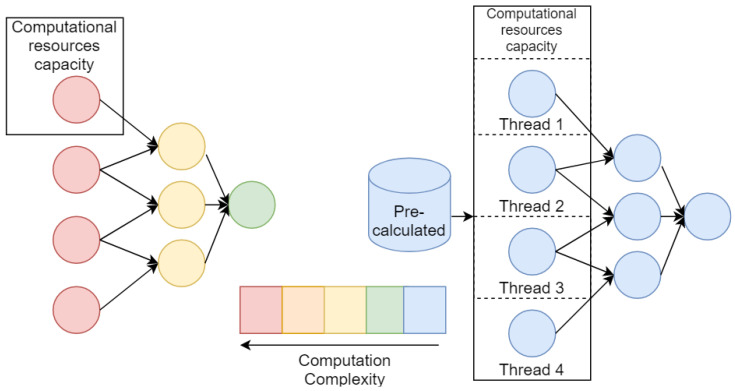
Setup that illustrates inefficiency of the parallel evolution implementation due to fitness function computation complexity.

Moreover, for the effective acceleration of the generative algorithm, we may take into account that most of the new composite models are based on nodes that are repeated numerously in the whole population. For such a case, we may provide storage for computed nodes and use them as results of pre-build models. The illustration of an ineffective and an effective parallelization setups described above is shown in the Figure 4.

The set of experiments that illustrates the problem raised in this issue and proposes the possible solutions is presented in Section 5.2.2.

*Issue 3. Co-Design of an Evolutionary Learning Algorithm and Computational Infrastructure*

In the frame of this research, the problem of co-design appears not only for the question of automatic creation of the computer model but also for the generative optimization algorithm itself. In Equation (8) we described co-design of generated model regarding the computational resources using estimation of model execution time $\;T_{M}$. Separate problem is adaptation of generative evolutionary algorithm regarding the computational resources and specific traits of the certain task. In formulation Equation (8) it was only accounted for by the restriction to the overall time $T_{gen}$ for model generation. However, the task can be formulated as search for generative evolutionary algorithm that is able to find the best model structure *M* in limited time $T_{gen}$. This task can be solved by optimization of hyper-parameters, evolutionary operators (and strategy of their usage) for generative optimization algorithm and formulated as meta-optimization problem over a set of possible algorithms *U* that are defined by a set of strategies B:

(13) $$U=\;\left\{u\left(B\right)\right\},\;B=\;\left\{b_{1:\left|B\right|}\right\},\;b=\langle H,R\rangle ,$$

(14) $$u^{*}=u\left(b^{*}\right)\;=\underset{b}{\arg\max}\;F\left(u\left(b\right)|T_{gen}\leq \tau_{g}\right),$$

where F is a meta-fitness function and each strategy *b* is defined by evolutionary operators R and hyper-parameters *H*. Evolutionary operators also my be described as hyper-parameters but here we subdivide them in separate entity R.

**Figure 5:**
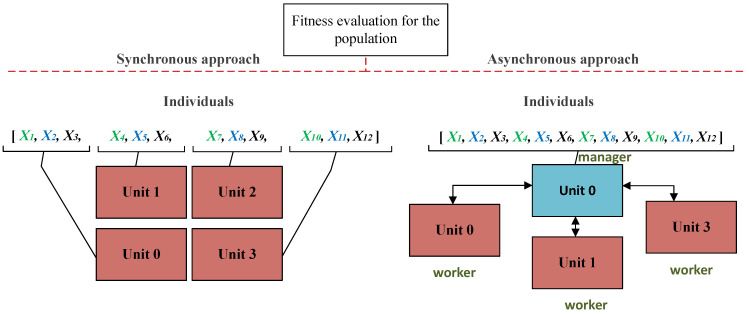
Approaches to the parallel calculation of fitness function with the evolutionary learning algorithm: (**a**) synchronously, each element of the population is processed at one node until all is processed (**b**) asynchronously, one of the nodes controls the calculations in other nodes.

For the model’s generative design task, the most expensive step usually refers to the evaluation of the fitness function value [6]. The calculation of the fitness function for the individuals of the evolutionary algorithm can be parallelized in different ways that are presented in Figure 5.

The described approaches can be used for the different variants of the computational environment used for the generation of the models. The practical application of the generated models with the complex structure almost always difficult because of the high computation complexity of the numerical model-based simulations.

There are several groups of models that can be separated by the simulation pipeline structure. For the data-driven model, the computational cost of the fitting (identification) stage is higher than for the simulation stage. For the equation-based numerical models with rigid structure, there is no implicit fitting stage, but the simulation can be very expensive. In practice, different parallelization strategies can be applied to improve simulation performance [39].

The set of experiments that provides the examples to the problem raised in this issue can be seen in Section 5.3.

*Issue 4. Computational Strategies for Identification of Graph M*

The problem of DAG $M=\langle S,E,\left\{a_{1:\left|S\right|}\right\}\rangle$ identification has two sides. First of all, the task of structural and parametric optimization of model *M* has exponential computational complexity with the growth of nodes number. Even if the functional structure $\langle S,E\rangle$ of the composite model is already identified, there is a computationally expensive problem of parameters $\left\{a_{1:\left|S\right|}\right\}$ (or hyperparameters in ML terms) tuning.

However, except for the computational intensity, there is a problem of searching the optimal set of values $\langle S^{*},E^{*},{\left\{a_{1:\left|S\right|}\right\}}^{*}\rangle$ in a space of high dimension (when chromosome has great length from tens to hundreds of values). This leads to unstable results of optimization algorithm because of the exponential growth of possible solutions in a combinatorial space (some parameters could be continuous but they are discretized and generally problem may be treated as combinatorial). One of the obvious ways for dealing with such a problem is local dimensionality reduction (or segmentation of the whole search space). This could be done with the application of various strategies. For example, we may simplify the task and search with generative algorithm only functional parts, and parameters (hyperparameters) may be optimized on the model execution stage (as discussed in Section 6). Such a way is economically profitable but we will get a result with lower fitness. An alternative variant is to introduce an approach for iterative segmentation of the domain space and greedy-like search on each batch (Section 5.4).

**Figure 6:**
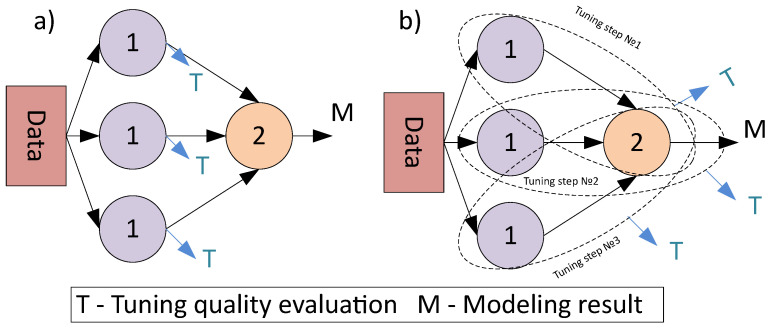
The different strategies of hyper-parameters tuning for the composite models: (**a**) individual tuning for each atomic model (**b**) the tuning of the composite model that uses secondary models to evaluate the tuning quality for the primary models.

Another point should be taken into account, the structure of DAG with directed edges and ordered nodes (composite model with primary and secondary nodes) leads to the necessity of introducing the sequential strategies for parameters tuning. Despite the tuning can be performed simultaneously with the structural learning, there is a common approach to apply it for the best candidates only [16]. Unlike the individual models tuning, the tuning of the composite models with graph-based structure can be performed with different strategies, that are represented in Figure 6.

The experiment that demonstrates the reduction of the search space for the composite model design by the application of the modified hyperparameters tuning strategy for the best individual is described in Section 5.4.

*Issue 5. Estimation of PM for Designed Models*

Analytic formulations of PM show expected execution time that is based on relation between approximate number of operations and computational performance of certain infrastructure configuration. The problem is to estimate this relation for all pairs from model structures $M=\langle S,E,\left\{a_{1:\left|A\right|}\right\}\rangle$ and computational resources $I=\left\{I_{q}\right\}$ with respect to input data *Z* because we need to make estimations of OML, ON and OC (depending on the class of models). Generally, there are two ways: (1) estimation of computational complexity (in O notation) for each model *M*, (2) empirical performance model (EPM) estimation of execution time for every specimen $\langle M,I,Z\rangle$. The first option gives us theoretically proved results, but this is hardly may be implemented in case of models’ generative design when we have too many specimens $\langle M,I,Z\rangle$. The second option is to make a set of experimental studies for specimens $\langle M,I,Z\rangle$ execution time measurements. However, in this case, we need to make a huge number of experiments before we start the algorithm of generative co-design and the problem statement becomes meaningless. To avoid numerous experiments, we may introduce estimation of EPM that consists of two steps. The first one is to estimate relation between time $\;\;T_{M}$ and volume of data *Z*: $T_{Num}^{\mathrm{PM}}\left(M,Z,I\right)\approx T_{Num}^{\mathrm{EPM}}\left(Z|M,I\right)$. To simplify identification of $T_{Num}^{\mathrm{EPM}}\left(Z|M,I\right)$, we would like to approximate this with a linear function with non-linear kernel $\psi \left(Z\right)$:

(15) $$T_{Num}^{\mathrm{EPM}}\left(Z\;\;\;|\;\;\;M,I\right)=\underset{w=1}{\sum^{W}}\;\omega_{w}\psi_{w}\left(Z\right),\;$$

where *W* is a number of components of linear function. The second step is to use value of $T_{Num}^{\mathrm{EPM}}\left(Z\;\;\;|\;\;\;M,I\right)$ to estimate relation between execution time and infrastructure *I*: $T_{Num}^{\mathrm{EPM}}\left(Z|M,I\right)\to T_{Num}^{\mathrm{EPM}}\left(I|M,Z\right)$. For this purpose we should make even a raw estimation of number of operations OML,ONandOC.

On the example of EPM for numerical model (Equation (10)) we can make the following assumptions:

(16) $$O\left(I\right)\approx 0,\;\;\;Const_{i}\left(I\right)\approx 0,\;V=mean_{i}\left(V_{i}\left(I\right)\right),\;\;ON=mean_{i}\left(ON_{i}\right),$$

(17) $$max_{i}\left[\frac{ON_{i}}{V_{i}\left(I\right)+Const_{i}\left(I\right)}\right]=\;mean_{i}\left[\frac{ON_{i}}{V_{i}\left(I\right)+Const_{i}\left(I\right)}\right],$$

and get the following transformations for raw estimation of overall number of operations nON with respect to *n* computational nodes:

(18) $$nON\left(M,Z\right)=nT_{Num}^{\mathrm{PM}}\left(M,Z,I\right)V\left(I\right),\;i\in \left[1:n\right].$$

It is worth nothing that the obvious way to improve accuracy of estimation nON is to use for experimental setup resources with characteristics of computational performance close to $V=mean_{i}\left(V_{i}\left(I\right)\right)$ and task partitioning close to $ON=mean_{i}\left(ON_{i}\right)$. Getting the estimation of nON and infrastructure parameters $V_{i}\left(I\right),\;Const_{i}\left(I\right),\;O\left(I\right)$ we may go to raw estimation:

(19) $$T_{Num}^{\mathrm{EPM}}\left(M,Z,I\right)=max_{i}\left[\frac{\alpha_{i}nON\left(M,Z\right)}{V_{i}\left(I\right)+Const_{i}\left(I\right)}\right]+O\left(I\right),$$

where $\alpha_{i}$ is coefficient for model partitioning. Similar transformations could be made for other models.

The experiments devoted to the identification of the empirical performance models for both atomic and composite models are provided in Section 5.5.

## 5. Experimental Studies

The proposed approaches to the co-design of generative models cannot be recognized as effective without experimental evaluation. To conduct the experiments, we constructed the computational environment that includes and hybrid cluster and several multiprocessor nodes that can be used to evaluate different benchmarks.

A set of experiments have been held with the algorithm of data-driven partial differential equation discovery to analyze its performance with different task setups. All experiments were conducted using the EPDE framework described in detail in [15].

The other set of experiments devoted to the automated design of the ML models was conducted using the open-source Fedot framework (https://github.com/nccr-itmo/FEDOT). The framework allows generating composite models using evolutionary approaches. The composite model generated by the framework can include different types of models [6]. The following parameters of the genetic algorithm were used during the experiments: maximum number of the generations in 20, number of the individuals in each population is 32, probability of mutation, probability of mutation is 0.8, probability of crossover is 0.8, maximum arity of the composite model is 4, maximum depth of the composite model is 3. More detailed setup is described in [40].

### 5.1. Choice of the Model Evaluation Algorithm

The first group of experiments is connected with the Issue 1 that describes the different aspects of numerical computation of designed models.

For example, the problem of data preprocessing for partial differential equations models, represented by the calculation of derivatives of the input field, is of the top priority for the correct operation of the algorithm: the incorrect selection of tools can lead to the increasing magnitudes of the noise, present in the input data, or get high values of numerical errors. The imprecise evaluation of equation factors can lead to cases, when the wrong structure has lower equation discrepancy (the difference between the selected right part term and the constructed left part) and, consequently, higher fitness values, than the correct governing equation.

However, the versatility of the numerical differentiation adds the second criterion on the board. The finite differences require a lot of expertise to choose and thus their automatic use is restricted since the choice of the finite difference scheme is not a trivial task that requires either a fine grid to reduce the error or choice of the particular scheme for the given problem. Both ways require extended time.

Artificial neural networks (ANN), used to approximate the initial data field, are an alternative to this approach, which can have a number of significant advantages. To get the fields of derivatives, we utilize the automatic differentiation, that is based on the approach, similar to the chain differentiation rule from the elementary calculus, and is able to combine the evaluated values of derivatives of a function, comprising the neural network to get the “correct” values of derivatives. In contrast to the previously used method of analytical differentiation of polynomials, the automatic differentiation is able to get mixed derivatives. From the performance point of view, the advantages of the artificial neural networks lie in the area of ease of parallelization of tensor calculations and the use of graphical processing units (GPU) for computation.

**Figure 7:**
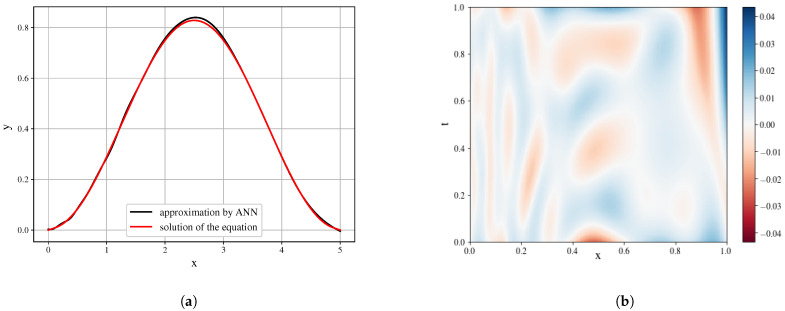
Comparison of the equation solution and its approximation by artificial neural networks (ANNs) for a time slice (**a**) and heatmap of the approximation error ($u_{\mathrm{approx}}-u_{\mathrm{true}}$) (**b**).

However, the task setup has a number of challenges in the approach to ANN training. First of all, the analyzed function is observed on a grid, therefore, we can have a rather limited set of training data. The interpolation approaches can alter the function, defining the field, and the derivatives, in that case, will represent the structure of the interpolating function. Next, the issue of the approximation quality remains unsolved. While the ANN can decently approximate the function of one variable (which is useful for tasks of ordinary differential equations discovery), on the multivariable problem statement the quality of the approximation is relatively low. The example of approximation is presented in Figure 7.

In the conducted experiments [41] we have used the artificial neural network with the following architecture: the ANN was comprised of 5 fully connected layers of 256, 512, 256, 128, 64 neurons with sigmoid activation function. As the input data, the values of the solution function for a wave equation ($u_{tt}=\alpha u_{xx}$), solved with the implicit finite-difference method, have been utilized. Due to the nature of the implemented solution method, the function values were obtained on the uniform grid. The training of ANN was done for a specified number of epochs (500 for the conducted experiments), when of the each epoch the training batch is randomly selected as a proportion of all points (0.8 of the total number of points). To obtain the derivatives, the automatic differentiation methods, implemented in the Tensorflow package are applied to the trained neural network.

**Figure 8:**
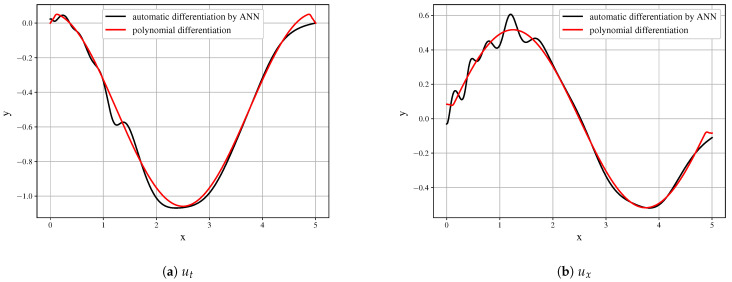
Comparison of derivatives obtained by polynomial differentiation and by symbolic regression for first time derivative (**a**) first spatial derivatives (**b**) for a time slice (t=50).

Even with the presented very good approximation of the original field, the first derivatives (Figure 8) are obtained with decent quality and may serve as the building blocks. However, it is seen that the derivative field is significantly biased.

**Figure 9:**
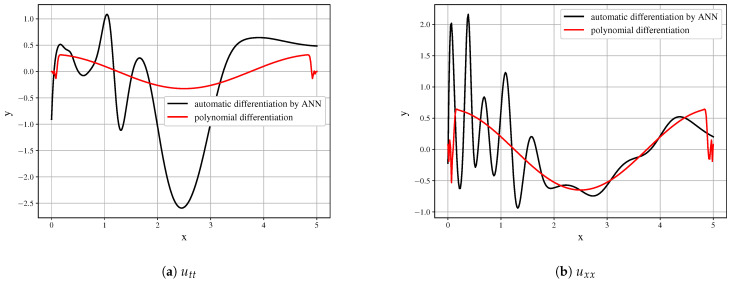
Comparison of derivatives obtained by polynomial differentiation and by symbolic regression for second time derivative (**a**) second spatial derivatives (**b**) for a time slice ( t=50).

Further differentiation amplifies the error. The higher-order derivatives shown in Figure 9 cannot be used as the building blocks of the model and do not represent the derivatives of the initial data field.

Both of the implemented differentiation techniques are affected by numerical errors, inevitable in the machine calculations, and contain errors, linked to the limitations of the method (for example, approximation errors). To evaluate the influence of the errors on the discovered equation structure, the experiments were conducted on simple ordinary differential Equation (ODE) (20) with solution function (21).

(20) $$L\left(t\right)=x\left(t\right)\sin t+\frac{dx}{dt}\cos t=1,$$

(21) x(t)=sint+Ccost.

**Figure 10:**
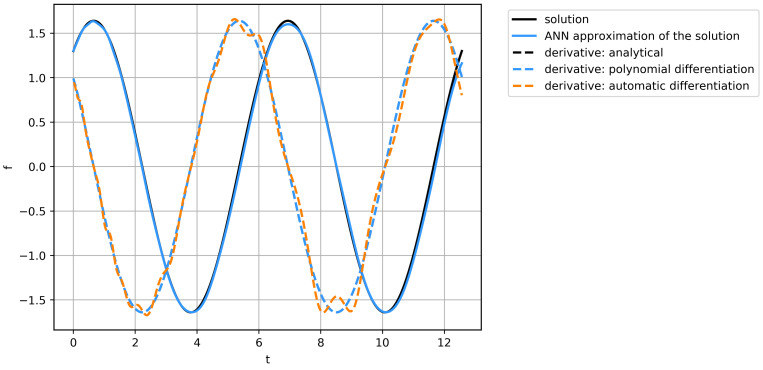
The solution of ODE from Equation (20), its approximation by neural network, and derivatives calculated by analytic, polynomial and automatic differentiation.

We have tried to rediscover the equation, based on data, obtained via analytical differentiation of function (21), application of polynomial differentiation, and with the derivative, calculated by automatic differentiation of fitted neural network. The series of function values and the derivatives are presented in Figure 10. Here, we can see, that the proposed ANN can decently approximate data; the analytical & polynomial differentiation obtains similar fields, while automatic differentiation algorithm may result in insignificant errors. 10 independent runs of the equation discovery algorithm have been performed for each derivative calculation method, and the results with the lowest errors have been compared. For the quality metric, the Mean Square Error of the vector, representing the discrepancy of the function $\bar{x}\left(t\right)$, which is the solution of discovered on data-driven equation M(t)=0 with aim of $\left|M\left(t\right)\right|\overset{}{\to }min$, evaluated on the nodes of the grid was used.

While all of the runs resulted in the successful discovery of governing equations, the issues with such equations are in the area of function parameters detection and calculating the correct coefficients of the equation. The best result was achieved on the data from analytical differentiation: $MSE=1.452\cdot 10^{-4}$. The polynomial differentiation got the similar quality $MSE=1.549\cdot 10^{-4}$, while the automatic differentiation achieved $MSE=3.236\cdot 10^{-4}$. It could be concluded, that in the case of first-order equations, the error of the differentiation has less order than all other errors and thus the fastest method for the given problem may be used. However, in the PDE case, it is complicated to use only first-order derivatives, whereas arbitrary ordinary differential equations may be represented as the system of the first-order equations.

### 5.2. Computationally Intensive Function Parallelization

#### 5.2.1. Parallelization of Generative Algorithm for PDE Discovery

The first experiment devoted to the parallelization of the atomic models’ computation using partial differential equations discovery case as an example. As shown in Figure 4, the parallelization of the evolutionary algorithm in some cases does not give significant speed improvement. In cases where atomic models are computationally expensive, it is expedient to try to reduce every node computation as much as possible.

The experiment [42] was dedicated to the selection of an optimal method of computational grid domain handling. It had been previously proven, that the conventional approach when we process the entire domain at once, was able to correctly discover the governing equation. However, with the increasing size of the domain, the calculations may take longer times. In this case parallelization of the evolutionary algorithm does not give speed-up on a given computational resources configuration, since the computation of a fitness function of a single gene takes the whole computational capacity.

To solve this issue, we have proposed a method of domain division into a set of spatial subdomains to reduce the computational complexity of a single gene. For each of these subdomains, the structure of the model in form of the differential equation is discovered, and the results are compared and combined, if the equation structures are similar: with insignificant differences in coefficients or the presence of terms with higher orders of smallness. The main algorithm for the subdomains is processed in a parallel manner due to the isolated method of domain processing: we do not examine any connections between domains until the final structure of the subdomains’ models is obtained.

The experiments to analyze the algorithm performance were conducted on the synthetic data: by defining the presence of a single governing equation, we exclude the issue of the existence of multiple underlying processes, described by different equations, in different parts of the studied domain. So, we have selected a solution of the wave equation with two spatial dimensions in Equation (22) for a square area, which was processed as one domain, and after that, into small fractions of subdomains.

(22) $$\frac{\partial^{2}u}{\partial t^{2}}=\frac{\partial^{2}u}{\partial x^{2}}+\frac{\partial^{2}u}{\partial y^{2}}.$$

However, that division has its downsides: smaller domains have less data, therefore, the disturbances (noise) in individual point will have a higher impact on the results. Furthermore, in realistic scenarios, the risks of deriving an equation, that describes a local process, increases with the decrease in domain size. The Pareto front, indicating the trade-off between the equation discrepancy and the time efficiency, could be utilized to find the parsimonious setup of the experiment. On the noiseless data (we assume, that the derivatives are calculated without the numerical error) even the data from a single point will correctly represent the equation. Therefore, the experiments must be held on the data with low, but significant noise levels.

**Figure 11:**
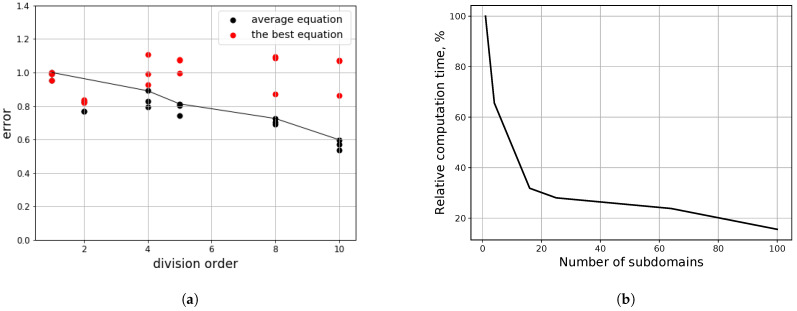
The results of the experiments on the divided domains. (**a**) evaluations of discovered equation quality for different division fractions along each axis (2× division represents division of domain into 4 square parts); (**b**) domain processing time (relative to the processing of entire domain) for subdomain number.

We have conducted the experiments with the partition of data (Figure 11), containing 80×80×80 values, divided by spatial axes in fractions from the set $\left\{\bar{1,10}\right\}$. The experiments were held with 10 independent runs on each of the setup (size of input data (number of subdomains, into which the domain was divided, and sparsity constant, which affects the number of terms of the equation).

The results of the test, presented in Figure 11, give insight into the consequences of the processing domain by parts. It can be noticed, that with the split of data into smaller portions, the qualities of the equations decrease due to the “overfitting” to the local noise. However, in this case, due to higher numerical errors near the boundaries of the studied domain, the base equation, derived from the full data, has its own errors. By dividing the area into smaller subdomains, we allow some of the equations to be trained on data with lower numerical errors and, therefore, have higher quality. The results, presented in the Figure 11b are obtained only for the iterations of the evolutionary algorithm of the equation discovery and do not represent the differences in time for other stages, such as preprocessing, or further modeling of the process.

We can conclude that the technique of separating the domain into lesser parts and processing them individually can be beneficial both for achieving speedup via parallelization of the calculations and avoiding equations, derived from the high error zones. In this case, such errors were primarily numerical, but in realistic applications, they can be attributed to the faulty measurements or prevalence of a different process in a local area.

#### 5.2.2. Reducing of the Computational Complexity of Composite Models

To execute the next set of experiments, we used the Fedot framework to build the composite ML models for classification and regression problems. The different open datasets were used as benchmarks that allow to analyze the efficiency of the generative design in various situations.

**Figure 12:**
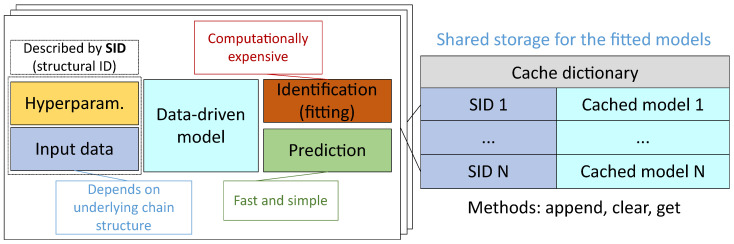
The structure of the multi-chain shared cache for the fitted composite models.

To improve the performance of the model building (this issue was noted in Issue 2), different approaches can be applied. First of all, caching techniques can be used. The cache can be represented as a dictionary with the topological description of the model position in the graph as a key and a fitted model as a value. Moreover, the fitted data preprocessor can be saved in cache together with the model. The common structure of the cache is represented in Figure 12.

**Figure 13:**
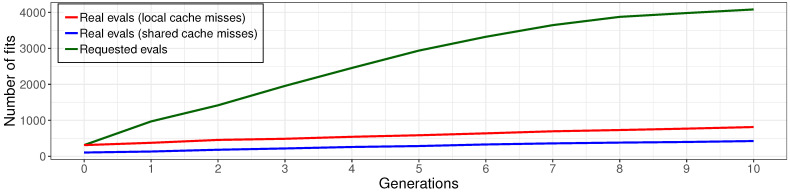
The total number model fit requests and the actually executed fits (cache misses) for the shared and local cache.

The results of the experiments with a different implementation of cache are described in Figure 13.

Local cache allows reducing the number of models fits up to five times against the non-cached variant. The effectiveness of the shared cache implementation is twice as high as that for the local cache.

The parallelization of the composite models building, fitting, and application also makes it possible to decrease the time devoted to the design stage. It can be achieved in different ways. First of all, the fitting and application of the atomic ML models can be parallelized using the features of the underlying framework (e.g., Scikit-learn, Keras, TensorFlow, etc [43]), since the atomic models can be very complex. However, this approach is more effective in the shared memory systems and it is hard to scale it to the distributed environments. Moreover, not all models can be efficiently parallelized in this way.

Then, the evolutionary algorithm that builds the composite model can be paralleled itself, since the fitness function for each individual can be calculated independently. To conduct the experiment, the classification benchmark based at the credit scoring problem (https://github.com/nccr-itmo/FEDOT/blob/master/cases/credit_scoring_problem.py) was used. The parameters of the evolutionary algorithm are the same as described at the beginning of the section.

**Figure 14:**
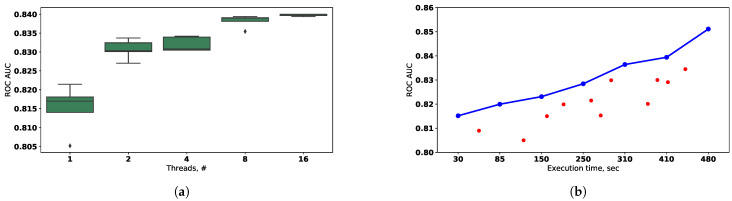
(**a**) The best achieved fitness value for the different computational configurations (represented as different number of parallel threads) used to evaluate the evolutionary algorithm on classification benchmark. The boxplots are build for the 10 independent runs. (**b**) Pareto frontier (blue) obtained for the classification benchmark in “execution time-model quality” subspace. The red points represent dominated individuals.

The obtained values of the fitness function for the classification problem are presented in Figure 14.

**Figure 15:**
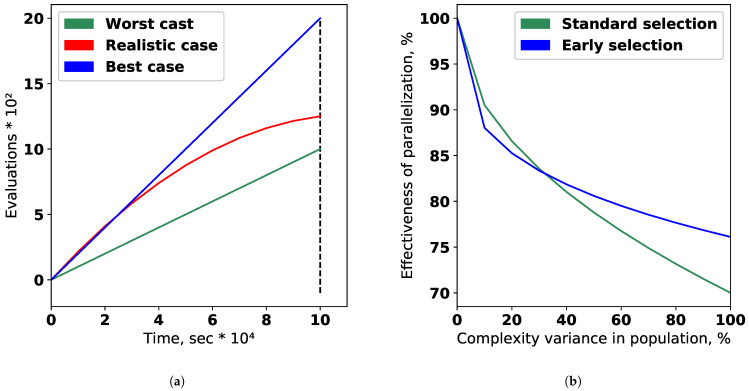
(**a**) The comparison of different scenarios of evolutionary optimization: best (ideal), realistic and worst cases (**b**) The conceptual dependence of the parallelization efficiency from the variance of the execution time in population for the different types of selection.

The effectiveness of the evolutionary algorithm parallelization depends on the variance of the composite models fitting time in the population. It is matters because the new population can not be formed until all individuals from the previous one are assessed. This problem is illustrated in Figure 15 for cases (a) and (b) that were evaluated with classification dataset and parameters of evolutionary algorithm described above. It can be noted that the modified selection scheme noted in (b) can be used to increase parallelization efficiency. The early selection, mutation, and crossover of the already processed individuals allow to start the processing of the next population before the previous population’s assessment is finished.

The same logic can be applied for the parallel fitting of the part of composite model graphs. It raises the problem of the importance of assessment for the structural subgraphs and the prediction of most promising candidate models before the final evaluation of the fitness function will be done.

### 5.3. Co-Design Strategies for the Evolutionary Learning Algorithm

**Figure 16:**
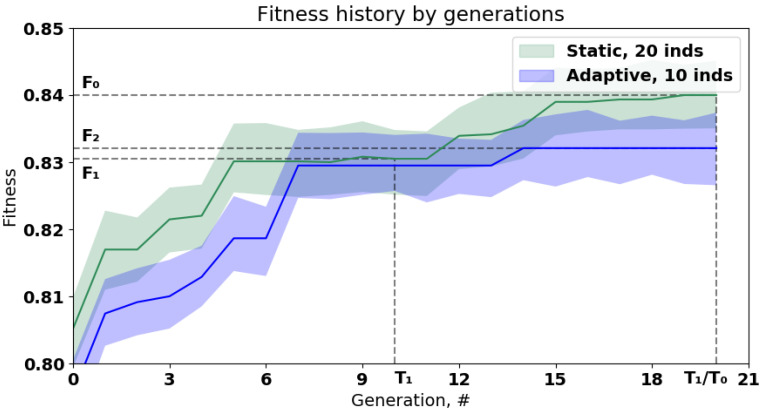
The comparison of different approaches to the evolutionary optimization of the composite models. The min-max intervals are built for the 10 independent runs. The green line represents the static optimization algorithm with 20 individuals in the population; the blue line represented the dynamic optimization algorithm with 10 individuals in the population. $T_{0}$, $T_{1}$ and $T_{2}$ are different real-time constraints, $F_{0}$, $F_{1}$ and $F_{2}$ are the values of fitness functions obtained with the corresponding constraints.

The co-design of the generative algorithm and the available infrastructure is an important issue (described in detail in the Issue 3) in the task of composite model optimization. The interesting case here is optimization under the pre-defined time constraints [44]. The experimental results obtained for the two different optimization strategies are presented in Figure 16. The classification problem was solved using the credit scoring problem (described above) as a benchmark for the classification task. The parameters of the evolutionary algorithm are the same as described at the beginning of the section. The fitness function value is based on ROC AUC measure and maximized during optimization.

The static strategy $S_{1}$ represents the evolutionary optimization with the fixed hyperparameters of the algorithm. The computational infrastructure used in the experiment makes it possible to evaluate the 20 generations with 20 individuals in the population with a time limit of $T_{0}$. This strategy allows finding the solution with the fitness function value $F_{0}$. However, if the time limit $T_{1}<T_{0}$ is taken into account, the static strategy allow to find the solution $S_{1}$ with the fitness function value $F_{1}$, where $F_{1}<F_{0}$.

Otherwise, the adaptive optimization strategy $S_{2}$, which takes the characteristics of the infrastructure to self-tune the parameters can be used. It allow to evaluate 20 generation with 10 individuals in a time limit $T_{1}$ and reach the fitness function value $F_{2}$. As can be seen, the $F_{1}<F_{2}<F_{0}$, so the better solution is found under the given time constraint.

### 5.4. Strategies for Optimization of Hyperparameters in Evolutionary Learning Algorithm

As it was noted in the issue described in Issue 4, the very large search space is a major problem in the generative design. To prove that it can be solved with the application of the specialized hyperparameters tuning strategies, a set of experiments was conducted.

As can be seen from Figure 6, the direct tuning strategy means that each atomic model is considered an autonomous model during tuning. The computational cost of the tuning is low in this case (since it is not necessary to fit all the models in a chain to estimate the quality metric), but the found set of parameters can be non-optimal. The composite model tuning allows to take into account the influence of the chain beyond the scope of an individual atomic model, but the cost is additional computations to tune all models. A pseudocode of an algorithm for composite model tuning is represented in Algorithm 1.
**Algorithm 1:** The simplified pseudocode of the composite models tuning algorithm illustrated in Figure 6b.
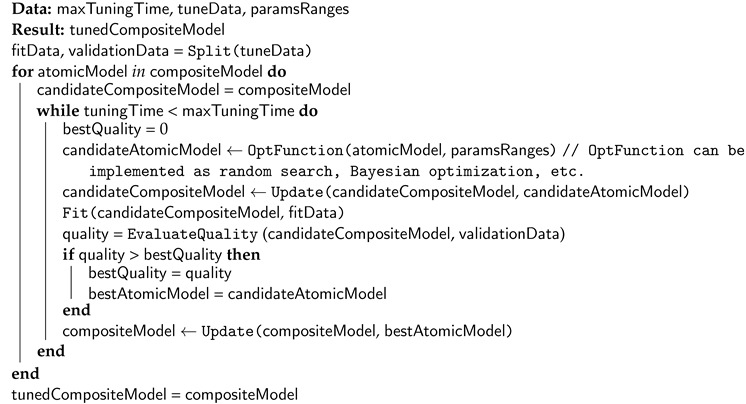


The results of the model-supported tuning of the composite models for the different regression problems obtained from PMLB benchmark suite (Available in the https://github.com/EpistasisLab/pmlb) are presented in Table 1. The self-developed toolbox that was used to run the experiments with PMLB and FEDOT is available in the open repository (https://github.com/ITMO-NSS-team/AutoML-benchmark). The applied tuning algorithm is based on a random search in a pre-defined range.

It can be seen that the hyperparameter optimization allow increasing the quality of the models in most cases.

### 5.5. Estimation of the Empirical Performance Models

The experiments for the performance models identification (this problem was raised in the issue described in Issue 5) were performed using the benchmark with a large number of features and observations in the sample. The benchmark is based on a classification task from the robotics field. It is quite a suitable example since there is a large number of tasks in this domain that can be performed on different computational resources from the embedded system to supercomputer in robotics. The analyzed task is devoted to the manipulator grasp stability prediction obtained from the Kaggle competition (https://www.kaggle.com/ugocupcic/grasping-dataset).

An experiment consists of grasping the ball, shaking it for a while, while computing grasp robustness. Multiple measurements are taken during a given experiment. Only one robustness value is associated though. The obtained dataset is balanced and has 50/50 stable and unstable grasps respectively.

The approximation of the EPM with simple regression models is a common way to analyze the performance of algorithms [46]. After the set of experiments, for the majority of considered models it was confirmed that the common regression surface of a single model EPM can be represented as a linear model. However, some considered models can be described better by another regression surface (see the quality measures for the different structures of EPM in Appendix A). One of them is a random forest model EPM. According to the structure of the Equation (9), these structures of EPM can be represented as follows:

(23) $$T^{\mathrm{EPM}}=\;\left\{\begin{array}{c} \Theta_{1}N_{obs}N_{feat}+\Theta_{2}N_{obs},\;\;for\;the\;\;common\;case\;\\ \frac{N_{obs}}{\Theta_{1}^{2}}+\frac{N_{obs}^{2}N_{feat}}{\Theta_{2}^{2}},\;\;specific\;case\;for\;random\;forest\;\; \end{array}\right.,$$

where $T^{\mathrm{EPM}}$—model fitting time estimation (represented in ms according to the scale of coefficients from Table 2), $N_{obs}$—number of observations in the sample, $N_{feat}$—number of features in the sample. The characteristics of the computational resources and hyperparameters of the model are considered as static in this case.

We applied the least squared errors (LSE) algorithm to (23) and obtained the Θ coefficients for the set of models that presented Table 2. The coefficient of determination $R^{2}$ is used to evaluate the quality of obtained performance models.

The application of the evolutionary optimization to the benchmark allows finding the optimal structure of the composite model for the specific problem. We demonstrate EPM constructing for the composite model which consists of logistic regression and random forest as a primary nodes and logistic regression as a secondary node. On the basis of (11), EPM for this composite model can be represented as follows:

(24) $$T_{Add}^{\mathrm{EPM}}=max\left(\Theta_{1,1}N_{obs}N_{feat}+\Theta_{2,1}N_{obs},\Theta_{1,2}^{1}N_{obs}N_{feat}+\Theta_{2,2}N_{obs}\right)+\frac{N_{obs}}{\Theta_{1,3}^{2}}+\frac{N_{obs}^{2}N_{feat}}{\Theta_{2,3}^{2}},$$

where $T_{Add}^{\mathrm{EMP}}$—composite model fitting time estimated by the additive EMP, $\Theta_{i},j$-*i* coefficient of *j* model type for EPM according to the Table 2.

**Figure 17:**
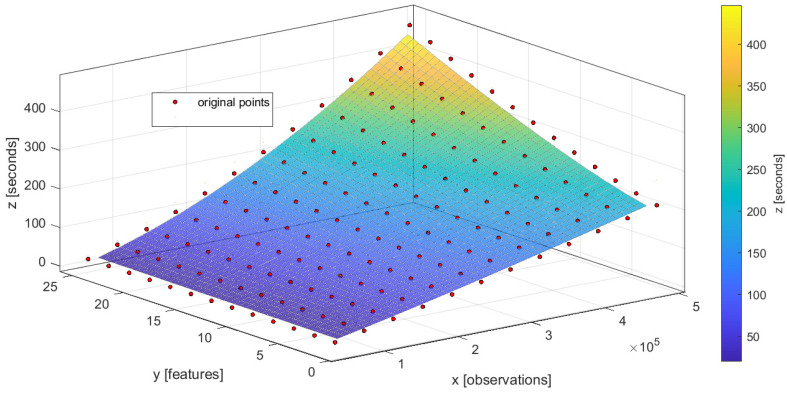
Predictions of the performance model that uses an additive approach for local empirical performance models (EPMs) of atomic models. The red points represent the real evaluations of the composite model as a part of validation.

The performance model for the composite model with three nodes (LR + RF = LR) is shown in Figure 17. The visualizations for the atomic models are available in Appendix A.

The RMSE (root-mean-squared-error) measure is used to evaluate the quality of chain EPM evaluation against real measurements. In this case, the obtained RMSE=21.3 s confirms the good quality of obtained estimation in an observed 0–400 seconds range.

## 6. Discussion and Future Works

In a wider sense co-design problem may be solved as an iterative procedure that includes additional tuning during the model execution stage and a cyclic closure (or re-building stage) with respect to time evolution. Re-building stage may be initiated by two types of events: (1) model error overcomes acceptable threshold $e_{c}$; (2) execution time overcomes acceptable threshold $\tau_{c}$. In this case a solution is to build the new model with respect to corrected set of structures $\tilde{S}$ and performance model $\;\;{\tilde{T}}_{M}$:

(25) $$p{{}^{\prime }}^{min}\left(M^{*},t\right)>\rho_{c},\;\;{T_{ex}}^{min}>\tau_{c},\;\;{\tilde{p}}^{min}\left(M^{**},t\right)=\max_{\tilde{M}}\;F^{\prime }\left(\tilde{M},t|\;\;\;{\tilde{T}}_{M}\leq \tau_{c},T_{gen}\leq \tau_{g}\right),\;$$

where *t* is a variable of real time and $\rho_{c}$ is a critical threshold for values of error function *E*. Such a problem is typical for models that are connected with a lifecycle of their prototype, e.g., models inside digital shadow for industrial system [47], weather forecasting models [48], etc.

Additional fitting of co-designed system may appear also on the level of model execution where classic scheduling approach may be blended with model tuning. Classic formulation of scheduling for resource intensive applications ${T_{ex}}^{min}\left(L^{*}\right)=\min_{A}\;G^{\prime }\left(L|M,I\right)$ is based on idea of optimization search for such algorithm $L^{*}$ that helps to provide minimal computation time ${T_{ex}}^{min}$ for model execution process through balanced schedules of workload on computation nodes. However, such approach is restricted by assumption of uniform performance models for all parts of application. In real cases performance of application may change dynamically in time and among functional parts. Thus, to reach more effective execution it is desirable to formulate optimization problem with respect to possibility of tuning model characteristics that influence on model performance:

(26) $${T_{ex}}^{max}\left({\left\{a_{1:\left|S\right|}\right\}}^{*},L^{*}\right)=\max_{a,L}\;G\left(M\left(\left\{a_{1:\left|S\right|}\right\}\right),L|I\right),\;\;M=S^{*},E^{*},\left\{a_{1:\left|S\right|}\right\}\;,\;\;\;\;L=\left\{L_{m}\right\},$$

where *G* is objective function that characterize expected time of model execution with respect to used scheduling algorithm *L* and model *M*. In the context of generative modeling problem on the stage of execution model *M* can be fully described as a set of model properties that consists of optimal model structure: optimal functions $S^{*}$ (from previous stage) and additional set of performance influential parameters $\left\{a_{1:\left|S\right|}\right\}$. Reminiscent approaches can be seen in several publications, e.g., [49].

## 7. Conclusions

In this paper, we aimed to highlight the different aspects of the creation of mathematical models using automated evolutionary learning approach. Such approach may be represented from the perspective of generative design and co-design for mathematical models. First of all, we formalize several actual and unsolved issues that exist in the field of generative design of mathematical models. They are devoted to different aspects: computational complexity, performance modeling, parallelization, interaction with the infrastructure, etc. The set of experiments was conducted as proof-of-concept solutions for every announced issue and obstacle. The composite ML models obtained by the FEDOT framework and differential equation-based models obtained by the EPDE framework were used as case studies. Finally, the common concepts of the co-design implementation were discussed.

## Figures and Tables

**Table 1:** The quality measures for the composite models after and before random search-based tuning of hyperparameters. The regression problems from PMLB suite [45] are used as benchmarks.

Benchmark Name	MSE without Tuning	MSE with Tuning	$R^{2}$ without Tuning	$R^{2}$ with Tuning
1203_BNG_pwLinear	8.213	0.102	0.592	0.935
197_cpu_act	5.928	7.457	0.98	0.975
215_2dplanes	1.007	0.001	0.947	1
228_elusage	126.755	0.862	0.524	0.996
294_satellite_image	0.464	0.591	0.905	0.953
4544_GeographicalOriginalofMusic	0.194	2.113	0.768	0.792
523_analcatdata_neavote	0.593	0.025	0.953	0.999
560_bodyfat	0.07	0.088	0.998	0.894
561_cpu	3412.46	0.083	0.937	0.91
564_fried	1.368	0.073	0.944	0.934

**Table 2:** The examples of coefficients for the different performance models.

ML Model	$\Theta_{1}\cdot 10^{4}$	$\Theta_{2}\cdot 10^{3}$	$R^{2}$
LDA	2.9790	3.1590	0.9983
QDA	1.9208	3.1012	0.9989
Naive Bayes for Bernoulli models	1.3440	3.3120	0.9986
Decision tree	31.110	4.1250	0.9846
PCA	3.1291	2.4174	0.9992
Logistic regression	9.3590	2.3900	0.9789
Random forest	$-94.42\cdot 10^{4}$	2.507$\cdot 10^{8}$	0.9279

**Figure A1:**
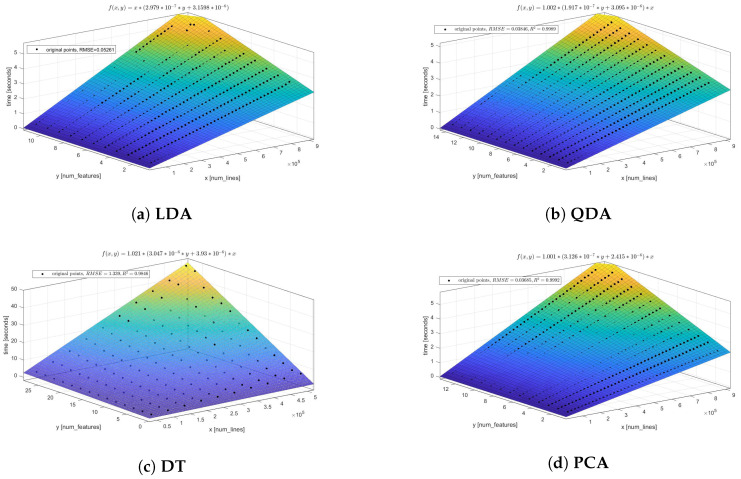

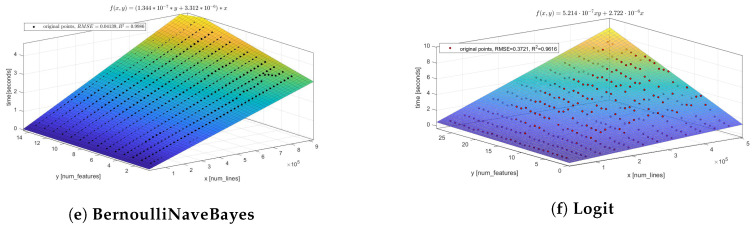
The empirical performance models for the different atomic models: LDA, QDA, Decision Tree (DT), PCA dimensionality reduction model, Bernoulli Naïve Bayes model, logistic regression. The heatmap represent the prediction of EPM and the black points are real measurements.

## Abbreviations

The following abbreviations are used in this manuscript:

AI	Artificial intelligence
ANN	Artificial neural network
AutoML	Automated machine learning
DAG	Directed acyclic graph
EPM	Empirical performance model
GPU	Graphics processing unit
ML	Machine learning
MSE	Mean squared error
NAS	Neural architecture search
ODE	Ordinary differential equation
PDE	Partial differential equation
PM	Performance model
$R^{2}$	Coefficient of determination
RMSE	Root mean square error
ROC AUC	Area under receiver operating characteristic curve

## Appendix A. Additional Details on the Empirical Performance Models Validation

The validation of different EPM for the set of the atomic models (that was noted in Table 2) is presented in Table A1. $R^{2}$ and RMSE metrics are used to compare the predictions of EPM and real measurements of the fitting time. The obtained results confirm that the linear EPM with two terms is most suitable for most of the ML models used in the experiments. However, the fitting time for some models (e.g., random forest) is represented better by the more specific EPM. The one-term EPM provides a lower quality than more complex analogs.

**Table A1:** Approximation errors for the different empirical performance models’ structures obtained for the atomic ML models. The best suitable structure is highlighted with bold.

Model	$\Theta_{1}N_{\mathrm{obs}}N_{\mathrm{feat}}$		$\Theta_{1}N_{\mathrm{obs}}N_{\mathrm{feat}}$ + $\Theta_{2}N_{\mathrm{obs}}$		$\frac{N_{\mathrm{obs}}}{\Theta_{1}^{2}}+\frac{N_{\mathrm{obs}}^{2}N_{\mathrm{feat}}}{\Theta_{2}^{2}}$	
	RMSE, s	$R^{2}$	RMSE, s	$R^{2}$	RMSE, s	$R^{2}$
LDA	0.35	0.92	**0.11**	**0.99**	0.66	0.74
QDA	0.75	0.57	**0.03**	**0.99**	0.93	0.36
Naive Bayes	0.82	0.42	**0.04**	**0.99**	0.961	0.21
Decision tree	1.48	0.98	**1.34**	**0.98**	3.49	0.89
PCA	0.28	0.78	**0.04**	**0.99**	0.28	0.95
Logit	0.54	0.91	**0.37**	**0.96**	0.95	0.75
Random forest	96.81	0.60	26.50	0.71	**21.36**	**0.92**

The visualization of the performance models predictions for the different cases is presented in Figure A1. It confirms that the selected EPMs allow estimating the fitting time quite reliably.
